# Deciphering the genetic control of gene expression following *Mycobacterium leprae* antigen stimulation

**DOI:** 10.1371/journal.pgen.1006952

**Published:** 2017-08-09

**Authors:** Jérémy Manry, Yohann Nédélec, Vinicius M. Fava, Aurélie Cobat, Marianna Orlova, Nguyen Van Thuc, Vu Hong Thai, Guillaume Laval, Luis B. Barreiro, Erwin Schurr

**Affiliations:** 1 Program in Infectious Diseases and Immunity in Global Health, The Research Institute of the McGill University Health Centre, Montreal, Quebec, Canada; 2 McGill International TB Centre, McGill University, Montreal, Quebec, Canada; 3 Departments of Medicine and Human Genetics, McGill University, Montreal, Quebec, Canada; 4 Department of Genetics, CHU Sainte-Justine Research Centre, Montreal, Quebec, Canada; 5 Department of Biochemistry, Faculty of Medicine, University of Montreal, Montreal, Quebec, Canada; 6 Laboratory of Human Genetics of Infectious Diseases, Necker Branch, Institut National de la Santé et de la Recherche Médicale U.1163, Paris, France; 7 Paris Descartes University, Imagine Institute, Paris, France; 8 Hospital for Dermato-Venerology, Ho Chi Minh City, Vietnam; 9 Institut Pasteur, Unit of Human Evolutionary Genetics, Department of Genomes and Genetics, Paris, France; 10 Centre National de la Recherche Scientifique, URA3012, Paris, France; 11 Center of Bioinformatics, Biostatistics and Integrative Biology, Institut Pasteur, Paris, France; 12 Department of Pediatrics, Faculty of Medicine, University of Montreal, Montreal, Quebec, Canada; Ospedale San Pietro Fatebenefratelli, ITALY

## Abstract

Leprosy is a human infectious disease caused by *Mycobacterium leprae*. A strong host genetic contribution to leprosy susceptibility is well established. However, the modulation of the transcriptional response to infection and the mechanism(s) of disease control are poorly understood. To address this gap in knowledge of leprosy pathogenicity, we conducted a genome-wide search for expression quantitative trait loci (eQTL) that are associated with transcript variation before and after stimulation with *M*. *leprae* sonicate in whole blood cells. We show that *M*. *leprae* antigen stimulation mainly triggered the upregulation of immune related genes and that a substantial proportion of the differential gene expression is genetically controlled. Indeed, using stringent criteria, we identified 318 genes displaying cis-eQTL at an FDR of 0.01, including 66 genes displaying response-eQTL (reQTL), i.e. cis-eQTL that showed significant evidence for interaction with the *M*. *leprae* stimulus. Such reQTL correspond to regulatory variations that affect the interaction between human whole blood cells and *M*. *leprae* sonicate and, thus, likely between the human host and *M*. *leprae* bacilli. We found that reQTL were significantly enriched among binding sites of transcription factors that are activated in response to infection, and that they were enriched among single nucleotide polymorphisms (SNPs) associated with susceptibility to leprosy *per se* and Type-I Reaction, and seven of them have been targeted by recent positive selection. Our study suggested that natural selection shaped our genomic diversity to face pathogen exposure including *M*. *leprae* infection.

## Author summary

Each year, 200,000 new leprosy cases are reported worldwide. While there is unambiguous evidence for a role of host genetics in leprosy pathogenesis, the mechanisms by which the human host fights the infection are poorly understood. Here, we highlight the search for naturally occurring genetic variations that modulate gene expression levels following exposure to sonicate of *Mycobacterium leprae*, the bacterium causing the disease. Because *M*. *leprae* is not cultivable and the genuine immune cells involved in the host response during infection are still unknown, we performed a genome-wide search for such genetic variations after stimulation of whole-blood from leprosy patients with *M*. *leprae* sonicate. This design allowed to provide a general framework for the genetic control of host responses to *M*. *leprae* and outlined the contribution of host genetics to leprosy pathogenesis. Among the *M*. *leprae*-dependent genetic regulators of gene expression levels there was an enrichment of variants (i) associated with leprosy, (ii) located in transcription factor binding sites and (iii) targeted by recent positive selection.

## Introduction

Leprosy is a human infectious disease caused by *Mycobacterium leprae*. Although curable, leprosy remains a major public health problem in sub-national regions of endemic countries [1]. The extremely low strain variability of *M*. *leprae* makes it a perfect model to access inter-individual differences in host responses to the bacillus since all leprosy patients are infected by virtually the same strain. Indeed, a strong host genetic contribution to leprosy susceptibility has been well established through the identification of leprosy susceptibility genes by both positional cloning (*PARK2*, *LTA*, *HLA-C*, *MRC1)* and candidate gene approaches (e.g. *TLR1*, *TNF*, *CUBN* and *NEBL)* [2–8]. Independently, genome-wide association studies (GWAS) allowed the identification of genes and pathways playing a crucial role in leprosy susceptibility such as genes of the HLA system and genes in the TNF pathway [9–11]. Most of the initially identified associations were replicated by subsequent studies [12–16]. Interestingly, two of the loci tagged by GWAS–*TNFSF8/TNFSF15*, *LRRK2 –*were later identified as risk factors for Type-I Reaction (T1R) which is the most frequent type of leprosy reaction, often leading to permanent disability [17–19]. Independently, a GWAS in Vietnamese and Brazilian populations identified a lncRNA as T1R risk factor [20]. Despite these advances in our understanding of the host genetic contribution to leprosy susceptibility, the modulation of the transcriptional response to infection and the mechanism of disease control are still poorly understood.

Immune responses against a particular infectious agent vary considerably between individuals and populations. Although a substantial proportion of these differences can be attributed to the environment, the contribution of host genetic factors is increasingly documented [21, 22]. Indeed, the contribution of host genetics to inter-individual differences in innate immune responsiveness has been recently demonstrated using expression quantitative trait loci (eQTL) mapping. This approach allows to identify associations between genotypes and variation of gene expression levels at baseline and in cells exposed to different immune stimuli or live infectious agents [21–26]. Employing human pathogens as trigger, these eQTL studies have identified a large number of host genetic variants that underlie variation in the host innate immune response to infection, including eQTL who can only be detected in either stimulated or non-stimulated conditions (i.e., response-cis-eQTL (reQTL)). Such reQTL reflect the direct interaction of genetic control elements with the pathogen and provide a model for gene-environment interactions [21–26].

Here, we used this approach to identify genetic variants that contribute to inter-individual variation in immune responses to *M*. *leprae* sonicate. To maximize the power for detection of eQTL, we enrolled subjects of a single ethnicity (Kinh) and susceptible background (leprosy patients) to decrease inter-individual variability. Since the genuine immune cell(s) involved in the host response against *M*. *leprae* infection are still unknown, we decided to focus on whole blood, which is a robust tissue for eQTL analysis [27–29]. In addition, the multitude of genes and pathways that play a role in leprosy susceptibility strongly suggested the involvement of multiple cell types further supporting the tissue approach to eQTL analysis[30]. By stimulating whole blood with soluble *M*. *leprae* antigen, we identified 318 genes harbouring cis-eQTL including 66 with reQTL. Hence, we provide a comprehensive scheme of the human host gene cis-regulation mechanisms in whole blood cells in response to *M*. *leprae* sonicate. To facilitate the use of our data by the research community, we integrated our results in a publicly available browser (http://immunpop.com/manry/eQTL), developed by [21].

## Results

### Immune response processes are enriched among upregulated genes

**Fig 1:**
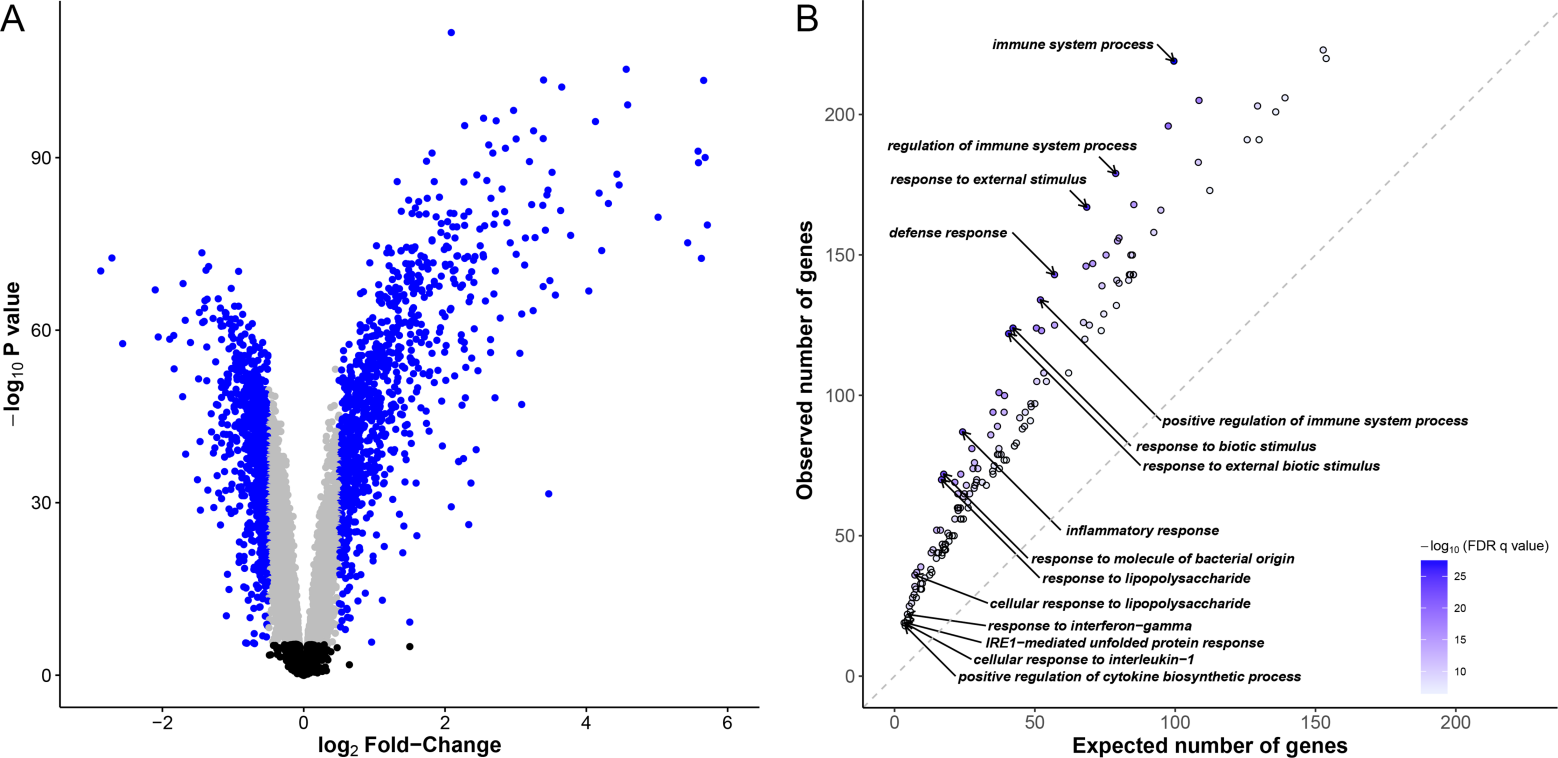
Identification of differentially expressed genes and functional characterization of immune responses to *M*. *leprae* sonicate. (A) Volcano plot showing differentially expressed genes after stimulation of whole-blood cells with *M*. *leprae* sonicate. The–log_10_ *P* values are plotted against the log_2_ FC. Black dots correspond to genes that are not differentially expressed, grey dots correspond to differentially expressed genes with a log_2_ FC < |0.5|, blue dots correspond to differentially expressed genes with a log_2_ |FC| > 0.5. (B) Gene ontology enrichment analysis for up-regulated genes (FDR q-value < 10^−6^) with a log_2_ FC > 0.5. Each dot corresponds to a gene ontology (GO) term. For example, the observed number of up-regulated genes belonging to the GO term “immune system process” is compared to the expected number of genes belonging to this same GO term among the genes which expression has been successfully measured. Only significant enrichments at an FDR q-value < 10^−6^ are included. Darker dots correspond to larger -log_10_(FDR q-values). The 10 GO terms displaying the largest -log_10_(FDR q-values) and the 5 GO terms displaying the highest enrichments are labelled. Of note, there is no gene ontology enrichment regarding down-regulated genes at this threshold.

We stimulated whole-blood cells from 51 Vietnamese subjects with *M*. *leprae* sonicate for 26–32 hours. We then extracted RNA from untreated and treated samples and characterized the genome-wide expression profiles in all samples leading to the analysis of 12,043 genes. Using a t-test, we found 6,675 differentially expressed genes after Bonferroni correction (*P* < 4.2x10^-6^), 2,338 being up-regulated, 4,337 being down-regulated after stimulation. Of note, 1,858 of those genes displayed an absolute log_2_ fold-change (log_2_|FC|) > 0.5 (Fig 1A). Gene ontology enrichment analysis confirmed that after stimulation the majority of up-regulated genes (74.5%) were related to immunity-related gene ontology (GO) terms such as “inflammatory response”, “response to molecule of bacterial origin” (false discovery rate (FDR) < 10^−26^). We found 177 GO terms with an FDR < 10^−6^, most of them (85.3%) were immune-related GO terms (Fig 1B, S1 Table). Prominently, among the upregulated genes were the Mendelian Susceptibility to Mycobacterial Disease genes of the *IFNG* pathway, including *IFNG*, *IFNGR2*, *STAT1*, *IRF8* and *IL12B* (“interferon-gamma-mediated signaling pathway”, FDR < 4.5x10^-7^). Conversely, there was no observed GO term enrichment with an FDR < 10^−6^ for down-regulated genes. Therefore, stimulation with *M*. *leprae* sonicate induced strong changes in the gene expression profile of whole blood cells, mainly dominated by the overexpression of genes involved in immune system processes and immune related functions.

### Identification and characterization of expression quantitative trait loci

After having identified differentially expressed genes, our goal was to identify genetic variants impacting gene expression levels. Following genome-wide genotyping and high quality imputation of 4,348,666 variants, we selected variants with a frequency higher than 10% in our sample that were located within a 200kb window of gene transcription start sites (TSS). Using these criteria, a total of 1,722,978 variants were tested for association with gene expression levels. To identify cis-eQTL, we used a linear regression model to assess association between the expression level of 12,043 autosomal genes and SNP genotypes independently in stimulated and non-stimulated cells. We identified a total of 318 genes displaying cis-eQTL at an FDR of 0.01 in either stimulated or non-stimulated samples (S1 Fig and S2 Table). Relaxing the FDR to 0.05 allowed us to identify 546 genes with cis-eQTL (S3 Table).

**Fig 2:**
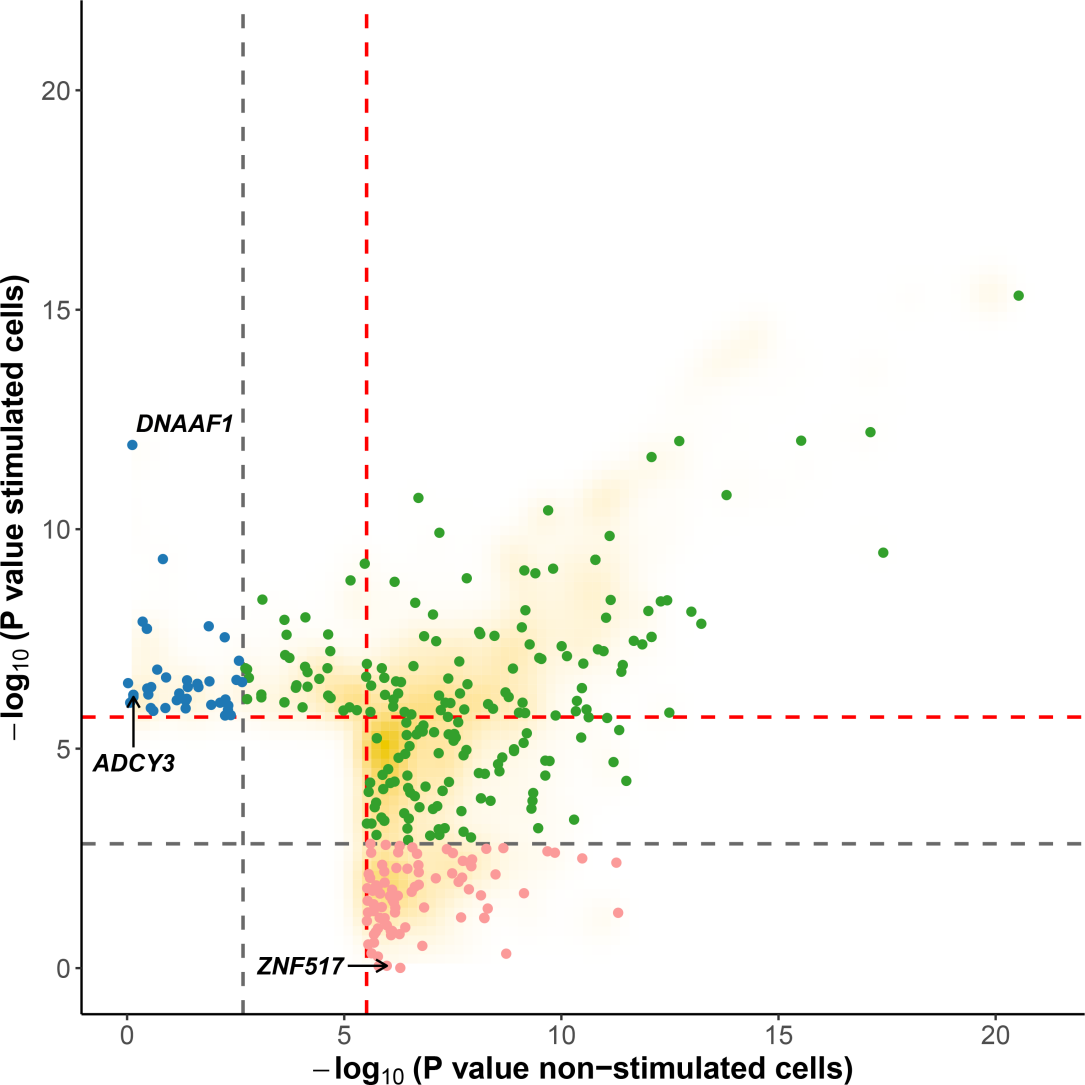
Evidence for cis-eQTL in stimulated *versus* non-stimulated cells. For each gene, we plotted the SNP with the lowest *P* value obtained under an additive model in one condition (stimulated or non-stimulated) against the *P* value obtained under the alternative condition. Red and grey dashed lines correspond to the 0.01 and to the 0.5 FDR to classify response eQTL (reQTL). Green dots are general cis-eQTL (found in both conditions). Blue dots are reQTL specific to cells stimulated with *M*. *leprae* sonicate while pink dots are reQTL specific to untreated cells. For this figure, reQTL are variants that exhibit a significant *P* value for genotype-phenotype association in one condition at an FDR of 0.01 and not in the other condition at an FDR of 0.5 (without taking the entire 200-kb tested regions per gene into account). The orange cloud corresponds to all the variants detected as being cis-eQTL at an FDR of 0.01.

**Fig 3:**
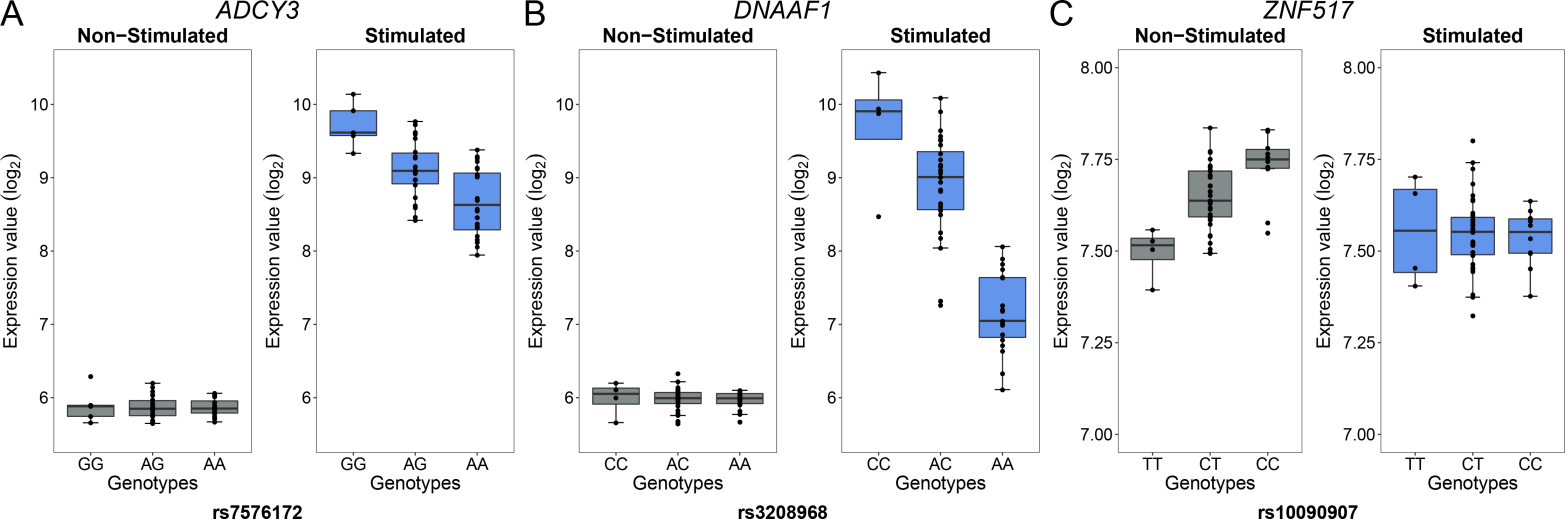
Examples of genes with reQTL likely to impact susceptibility to leprosy. Examples of three reQTL among genes found only in *M*. *leprae* sonicate stimulated cells or non-stimulated cells. For each gene ((A) *ADCY3*, (B) *DNAAF1* and (C) *ZNF517*): the left panel corresponds to the expression of the gene in non-stimulated cells while the right panel depicts expression of the gene in stimulated cells. The gene identity is indicated above each pair of graphs. The gene expression level in log_2_ scale (y-axis) is plotted for each genotype (x-axis). Of note, reQTL for the *ADCY3* and *DNAAF1* genes have been found by other studies using distinct pathogens or molecules as stimuli, while the reQTL for *ZNF517* is a newly identified reQTL [21, 22, 24, 26]. *ADCY3* is among the most upregulated genes after stimulation with *M*. *leprae* antigens and has been identified as part of the T1R gene set signature identified by Orlova *et al*. [32]. The reQTL for *DNAAF1* displays the strongest *P* value among the reQTL we identified.

In the context of the cellular response against *M*. *leprae* sonicate, cis-eQTL that are present only before or only after stimulation have a more direct role in modulating the host response against *M*. *leprae* because they are impacted by the mycobacterial sonicate. To determine the proportion of eQTL specific to each condition, we used a continuous measure π_1_ [31], which allowed us to estimate the proportion of eQTL that are shared in stimulated cells and non-stimulated cells. When considering only the best eQTL signal per gene (the variant with the lowest *P* value, S2 Table), we obtained π_1_ = 0.905 and 0.935 for stimulated and non-stimulated cells, respectively, suggesting that although most eQTL are shared, a non-negligible fraction are only detected in stimulated or non-stimulated cells. To identify which variants are responsible for such specific eQTL, we searched for “response cis-eQTL” (reQTL) as defined by Barreiro *et al*., using stringent criteria to minimize the probability of false positives (see Material & Methods) [23]. We found a total of 66 genes displaying reQTL. A total of 20 genes were associated with an eQTL only in stimulated cells and 46 only in non-stimulated cells (Figs 2 and 3, S4 Table). Among them, *ADCY3* is among the most upregulated genes after stimulation with *M*. *leprae* antigens and has been identified as part of the T1R gene set signature identified by Orlova *et al*. [32] (Fig 3A). To assess whether the reQTL identified here were preferentially located in transcription factor binding sites, we used the annotation provided by HaploReg v4 and resampled the position of all reQTL across the tested regions (200kb around TSS). We found a significant enrichment of reQTL located in binding sites for 54 transcription factors (Resampling *P* values between 5x10^-6^ and 0.042 and a fold-change ranging from 1.66 to 4.64). Four transcription factors tagged by this analysis are involved in immune-mediated diseases such as systemic lupus erythematosus, inflammatory bowel disease and multiple sclerosis (CFOS, ELF1, ETS1 and NFKB; S5 Table). In addition, we evaluated the effect of reQTL on TFBS using the SNP2TFBS tool (http://ccg.vital-it.ch/snp2tfbs) [33], which predicts a change in TF binding based on position weight matrices (PWM). This approach allowed us to identify seven TF (Mafb, E2F4, Zfx, E2F1, PPARG_RXRA, ELK1 and NR3C1) for which binding sites altered by a reQTL were significantly enriched above genome-wide expectation (S6 Table). Among those, NR3C1 (also know as the glucocorticoid receptor GR) is involved in inflammatory responses [34]. Of note, E2F1 and NR3C1 binding sites were enriched among reQTL in both methods.

### Leprosy *per se* and Type-I Reaction GWAS hits are enriched for reQTL

Our group recently conducted two GWA scans for leprosy *per se* (Cobat *et al*, in preparation) and leprosy T1R [20] in the Vietnamese population. To investigate a possible contribution of cis-eQTL to the genetic control of these phenotypes, we searched for an enrichment of cis-eQTL displaying GWAS *P* values below 0.05. When focusing on association under the best genetic model for each SNP (recessive, additive or dominant), the leprosy *per se* and the T1R GWAS exhibited 8.2% (951,685 SNP out of a total of 11,614,395 tested SNPs) and 7.7% (876,126 SNP out of a total of 11,342,181 tested SNPs) of SNPs with *P* value < 0.05, respectively. An enrichment of cis-eQTL among those nominally associated SNPs would be indicative of a role for the genetic control of transcript levels in both leprosy and T1R. To avoid any bias due to linkage disequilibrium between eQTL for each gene, we only considered the “best” eQTL per gene, i.e. the one displaying the lowest *P* value. We thus extracted the GWAS *P* values for each “best” cis-eQTL from both GWAS studies and identified the proportions of cis-eQTL with GWAS *P* values < 0.05. Using such a stringent criterion, a total of 9.1% of cis-eQTL (1.1-fold enrichment, resampling *P* value = 0.24) displayed a *P* value below 0.05 for association with leprosy *per se*. However, 10.4% of cis-eQTL (1.3-fold enrichment, resampling *P* value = 0.03) met this condition for association with T1R. Likewise, reQTL were enriched among SNPs associated with low GWAS-*P* values for both GWAS (1.7-fold enrichment, *P* value = 0.04 for leprosy *per se* and 2.4-fold enrichment, *P* value = 0.001 for T1R). To confirm these results considering all the cis-eQTL and reQTL, we used the GARFIELD software [35], which integrates the LD structure between each eQTL or reQTL, their frequencies, and their distance to the nearest TSS. Under these more stringent conditions, we observed a trend of association for reQTL among T1R and leprosy per se GWAS hits (P = 0.07 and P = 0.11, respectively). Hence, cis-eQTL identified in our study are candidates for affecting susceptibility to T1R, while reQTL are candidates for affecting both susceptibility to leprosy *per se* and T1R.

### Recent positive selection targeted reQTL

Since eQTL have been shown to be targeted by recent selection in humans, we sought to determine the extent to which natural selection targeted cis-eQTL and reQTL detected in our study [36]. As the individuals recruited for our study were all Kinh in Ho Chi Minh City, Vietnam (KHV) people, we used whole-genome sequences of the 1,000 Genomes Project Phase III of the same KHV population (101 samples) [37]. All samples clustered with the KHV population from the 1000 genomes project (S2 Fig). We calculated the normalized Derived Intra-allelic Nucleotide Diversity test (DIND), which is used to detect ongoing selective sweeps within a population, on each SNP found in the KHV population of the 1,000 Genomes Project and obtained ranked *P* values for this test [38–40]. This test is based on the rationale that a derived allele (i.e. the allele specific to the human lineage) under positive selection present at high frequency in the population should display lower levels of nucleotide diversity at linked sites than expected. We then extracted the ranked *P* value for each “best” eQTL and reQTL of our study. We compared our results with genome-wide expectations by resampling and found a significant enrichment of DIND ranked *P* values below 0.05 among cis-eQTL and a similar trend for reQTL (Resampling *P* values = 0.025 and 0.168 for an enrichment of 1.7-fold and 1.85-fold, respectively). These results are consistent with two recent studies using a similar approach [21, 22].

We also calculated the global *F*_ST,_ which is a measure of population differentiation between all the populations of the 1000 Genomes Project, for each cis-eQTL and reQTL. High *F*_ST_ values (those closer to 1) are indicative of positive selection. We found 17 eQTL (including two reQTL) displaying an *F*_ST_ value above the 95^th^ percentile of the *F*_ST_ distribution against the minor allele frequency. Overall, considering both DIND and *F*_ST_, we found 35 cis-eQTL (including 7 reQTL for the *DUSP16*, *CABLES2*, *ODF2L*, *UBA7*, *GFM1*, *CORO1C*, *CEP192* genes) targeted by recent positive selection (S7 Table). Finally, we searched for cis-eQTL exhibiting both a signal of recent positive selection and a GWAS *P* value < 0.05. Interestingly, we found three reQTL controlling the expression of the *GFM1*, *CORO1C* and *CEP192* genes that also displayed significant DIND values (S7 Table). While the reQTL found for *CEP192* exhibits a *P* value for association below 5% in both GWAS, *CORO1C* exhibits such a *P* value for the leprosy GWAS only, and *GFM1* for the T1R GWAS only, suggesting these reQTL as targeted by distinct selective pressures and as important players during recent human evolution (for a summary flowchart of the results, see S3 Fig).

## Discussion

In this study, we demonstrated the impact of cis-eQTL and in particular of reQTL during *M*. *leprae* antigens stimulation of human blood. While reQTL have been found for other infections [21–26], the newly described reQTL directly reflect the interaction of the genetic background of the human host with *M*. *leprae* antigens. The variants composing reQTL represent promising candidates for mediating resistance and/or susceptibility to *M*. *leprae* infection. We note, however, that the reQTL for 66 genes found in our study may not be exclusive to *M*. *leprae* infection. It is possible that the genes tagged are more broadly involved in host responses to infectious pathogens. For example, when comparing our results with reQTL observed by Barreiro *et al*. before or after *M*. *tuberculosis* infection of dendritic cells, we found an overlap of one reQTL for *FOXJ2* [23]. This reQTL had been found after *Salmonella typhimurium* but not *Listeria monocytogenes* infection of macrophages, and after Influenza A virus infection of monocytes suggesting this reQTL as being cell type- and stimulus- dependent. In general terms, reQTL are exquisite indicators for host gene-pathogen interactions. Compared to eQTL, reQTL are expected to display an increased pathogen-specificity, which is consistent with the small overlap of reQTL detected here and in *M*. *tuberculosis* infected dendritic cells [23]. The latter interpretation is in line with the results of genetic studies for leprosy and tuberculosis susceptibility that detected a limited overlap in the genetic control of both diseases [12]. These findings imply that any evolutionary constraints exerted by TB, a disease with high childhood mortality, on the genetic control of gene expression levels may not be reflected in the human immune response to *M*. *leprae*.

Genes that alter their expression levels following stimulation with *M*. *leprae* sonicate are part of the host response against *M*. *leprae* bacilli. Consequently, cis-eQTL and reQTL for those genes are candidates for being genetic modifiers of human vulnerability against *M*. *leprae* and thus possible targets of natural selection. A large number of significantly differentially expressed genes were detected in our study. However, given our sample size (51 samples) and the high dose of *M*. *leprae* sonicate used for stimulation (equivalent to an estimated MOI of 50:1), we expected to detect a large proportion of differentially expressed genes in whole blood samples. Additional power calculations confirmed that power was nearly 100% to detect log_2_ |FC| larger than 0.2, the threshold employed in our study being 0.5 (S5 Fig). We employed an evolutionary genetics approach to reveal which cis-eQTL and reQTL were targeted by positive selection. Previous studies had suggested that eQTL contributing to inter-individual variation in immune responses to pathogens or immune stimuli were enriched among SNPs targeted by recent selection [21, 22]. A possible limitation of our approach was that we focused on the “best” eQTL per gene, which represents a high degree of stringency. Indeed, the “best” eQTL is not more likely than other SNPs of the same locus to be the one targeted by natural selection and/or the causal one. Even if the LD is strong between the SNPs of an eQTL, neutrality tests can vary between those SNPs. Thus, it is likely that we underestimated the number of eQTL targeted by positive selection, and the deduced enrichment represents a conservative approximation of the true action of natural selection on eQTL. Moreover, several variants may control synergistically the expression of a gene and taking only the “best” variant for enrichment analyses may be restrictive. Despite these limitations, we identified an enrichment of cis-eQTL under positive selection in the Kinh in Ho Chi Minh City, Vietnam, population. It is not known if this enrichment reflects the selective action of leprosy or is the result of evolutionary forces acting on host responses shared with other pathogens.

Since reQTL are likely to have a direct role in the response against *M*. *leprae* we were particularly interested in signatures of selection for such SNPs. Among the seven genes with reQTL targeted by recent positive selection (*DUSP16*, *CABLES2*, *ODF2L*, *UBA7*, *GFM1*, *CORO1C*, *CEP192*), only *UBA7* displays a clear role in the immune system. Overall, *UBA7* is downregulated after stimulation (FC = -0.34). However, the selected alleles are associated with high expression of *UBA7* in stimulated cells (*P* values for the genotype-gene expression association: 0.05 and 1.2x10^-6^ in non-stimulated cells and in stimulated cells, respectively). A decrease in expression of *UBA7* might result in the diminution of the conjugation with ISG15, which has been shown to be important to fulfil its antiviral activity [41–44]. More importantly, ISG15 has been shown to be a key effector molecule in anti-mycobacterial immunity, a role that is strongly supported by our present finding [45]. The downregulation of *UBA7* after stimulation with *M*. *leprae* sonicate might reflect a means of the pathogen to circumvent human immunity while the reQTL possibly illustrates how host evolution is counteracting pathogen manipulation. To what extent changes in *UBA7* expression levels impact ISG15-dependent intracellular accumulation of the IFN-α/β regulator USP18 is unknown [46].

In previous studies, a link between positively selected variants and disease resistance has been shown and an enrichment for signals of recent positive selection has been found among SNPs associated with immunity-related phenotypes such as immune-mediated and infectious diseases [38, 40, 47, 48]. While natural selection in humans is acting against infectious diseases and in favour of strong protective immune responses, excessive immune reactivity may cause immune-mediated host tissue pathology [47]. In leprosy, protective and host pathological immunity are temporally separated since nerve damage due to T1R usually occurs after the onset of *M*. *leprae* infection and clinical disease. We have previously shown distinct genetic control of T1R and leprosy [17, 20]. By dovetailing the results of the eQTL analysis with the genetic control of leprosy and T1R we were able to assign genetic controllers of *M*. *leprae* host responses to either the protective (mycobacterial clearance) or the host damaging arm of the immune response (excessive inflammatory reaction characteristic for T1R). Identifying cis-eQTL and reQTL that displayed association with only one of these two phenotypes reinforced the differences in their aetiology and revealed that distinct modifiers of gene expression act on the protective and the host damaging part of the immune response. Interestingly, the integration of the functional genomics approach and GWAS data with the evolutionary genetics approach allowed us to identify reQTL for three genes, *GFM1*, *CORO1C* and *CEP192*, that likely have a major role against infection with *M*. *leprae* and/or T1R. Among these three genes, *GFM1* is of particular interest. It has been suggested that *M*. *leprae* represses mitochondrial genes in Schwann cells [49]. It is plausible that this repression is triggered by a deregulation of *GFM1* by *M*. *leprae* which might disturb translation of mitochondrial RNAs. Of note, among the GO term enrichments for upregulated genes after *M*. *leprae* antigen stimulation, we found the “release of cytochrome c from mitochondria” GO term (FDR of 3x10^-3^) However, the specific role of *GFM1* in leprosy pathogenesis needs to be determined by functional studies. Given their implication by three distinct lines of experimental evidence, the role of these three genes in leprosy pathogenesis deserves more careful scrutiny.

## Materials and methods

### Ethics statement

The study was conducted according to the principles expressed in the declaration of Helsinki. Written informed consent was obtained for all adult subjects participating in the study. All minors assented to the study, and a parent or guardian provided written informed consent on their behalf. This study was approved by the regulatory authorities and ethics committees in Ho Chi Minh City, Vietnam (No. 4933/UBND-VX), and the Research Ethics Board at the RI-MUHC in Montreal QC, Canada (REC98-041).

### Subjects

A total of 51 unrelated individuals from Vietnam diagnosed with borderline leprosy were recruited, including 41 males and 10 females, with a mean age of 27 years old (range 9 to 41 years old) [32]. A total of 40 of these samples had been part of the study by Orlova *et al*. [32]. We performed a principal component (PC) analysis to check for population structure.

### Whole blood assay and RNA extraction

A total of 20mL of whole blood were obtained from each subject by venipuncture in EDTA vacutainers. A 10mL aliquot was stimulated with *M*. *leprae* sonicate at a concentration of 20ug/mL, the other 10mL remained untreated for 26–32 hours at 37°C, 5% CO_2_ as described by Orlova *et al*. [32]. Stimulations of whole blood samples were done in triplicate wells for stimulated cells and unstimulated controls. For RNA extraction, each set of wells was combined in a single batch. We decided for a relatively long stimulation time since faster host responses did not reveal pathogen-specificity of their genetic control [50]. Total RNA was then extracted employing a modified protocol of the LeukoLOCK RNA extraction kit (Ambion, CA, USA) and cleaned with the RNAeasy kit (Qiagen, Germany) as described by Orlova *et al*. [32]. All 102 samples passed quality control by BioAnalyzer (Agilent) and showed RNA Integrity Numbers above 8.5, indicating good RNA quality.

### Gene expression data analysis

The 102 RNA samples were labelled using the Illumina TotalPrep RNA Amplification Kit from Ambion and hybridized to Illumina HumanHT-12 v4 Expression BeadChips and screened for 47,323 probes. All samples were randomly assigned to chips for hybridization. Gene expression levels were determined by a single microarray experiment for each sample.

Using the lumi package available in R [51], raw data were subjected to variance-stabilization transformation (VST) and quantile normalization. Only probes in autosomes, uniquely mapped and expressed above background noise (*P* value < 0.05) in at least 3 individuals were kept. Probes that did not match to any unique Ensembl gene and Hugo ID were excluded. For genes with several probes, to prevent spurious signals, we kept the median expression value. It is worth noting that we decided not to remove probes mapping to regions with SNPs in the KHV population following the recommendations by Schurmann *et al*. [52]. Supporting a weak effect of SNPs in probes, only 16 eQTL-associated genes were targeted for one probe overlapping a SNP position. As we cannot rule out the possibility that these 16 eQTL associations are spurious, they were flagged in the S2 Table. This led us to the analysis of 12,043 genes. The gene expression data were then analyzed with a linear model with a fixed effect for the treatment, and integrating age, sex, and the duration of stimulation as covariates. Gene expression levels of stimulated samples were also adjusted on dose since for technical reasons not all samples received the exact same dose. Principal component analysis showing the effect of each covariable and their corrections are shown in the S4 Fig. In addition, no impact of chip batch on results was observed.

### Identifying differentially expressed genes and gene ontology enrichment analysis

With R, we used a linear model to perform a t test to capture the effects of the stimulation with *M*. *leprae* sonicate and calculated the log_2_(FC) for each gene. Genes were considered as differentially expressed after Bonferroni correction. The t test has been shown to be as powerful as the moderated t test for sample sizes higher than 15 samples [53]. In accordance, the comparison of our method (t test and Bonferroni correction) with the more standard limma method (moderated t test and Benjamini-Hochberg correction [54]) identified 1858/1860 (99.9%) of the same genes differentially expressed with a log_2_|FC| > 0.5. There was no difference in differentially expressed genes when a paired sample analysis was applied considering differentially expressed genes at |FC| > 0.5 (adjusted P values < 2.8x10^-6^). Power analysis was performed using the package sizepower implemented in R [55]. A |FC| of 0.5 has been used throughout the manuscript unless stated otherwise. We used GOrilla to test for enrichment of biological processes, molecular functions and cellular components among differentially expressed genes after stimulation considering upregulated genes and downregulated genes separately, against the 12,043 tested genes for association [56]. *P* values were obtained using a hypergeometric model and the false discovery rate (FDR) was controlled by the Benjamini-Hochberg method [57].

### Genotype-phenotype association analysis

DNA from the blood donors was extracted using the Nucleon BACC 2 kit (GE HealthCare) and genotyping was performed using Illumina`s Human660W-Quad BeadChip array. After standard filtration methods (no missing data, Hardy-Weinberg *P* value < 10^−4^), we kept 459,703 SNPs (S1 Dataset). Non-genotyped SNPs were imputed using SHAPEIT and IMPUTE2 and the 1000 genomes Phase I v3 dataset containing 1092 individuals as the reference panel, leading to 3,888,963 variants with an imputation information ≥ 0.5 [58, 59]. Each gene expression value and genotypes at variants located within a 200kb window centered on the gene’s transcription start site were tested for association. A total of 1,722,978 variants were thus tested for association. The FDR was estimated by permuting 10 times the phenotypes (expression data), and comparing these 10 distributions to the observed one.

To increase power to detect cis-eQTL as previously described by Barreiro *et al*., we determined the PCs of the stimulated and non-stimulated expression data and regressed out the first 8 and 7 PCs from the stimulated and non-stimulated data, respectively [23]. The numbers of PCs to regress out were chosen because they led to the identification of the highest number of cis-eQTL (gain of more than 108% and 96% for stimulated and non-stimulated data, respectively), while conserving most of the originally found cis-eQTL (>86% and >88% of the original cis-eQTL set were conserved after PC removal for the stimulated and non-stimulated data, respectively). We found 318 cis-eQTL (composed by 13,825 variants) at an FDR of 0.01 with the 200kb window and 546 (22,449 variants) at an FDR of 0.05 (S2 and S3 Tables). For more relaxed FDR, the reader can access the http://immunpop.com/manry/eQTL website. To identify whether some eQTL were specific to one condition or the two conditions, we used the Storey and Tibshirani q-value approach implemented in the qvalue package available in R to estimate π_0_, the proportion of variants that are truly not eQTL in one condition while eQTL in the other [31]. Concerning the identification of reQTL, a variant was defined as such by using a two-step FDR cut-off as described by Barreiro *et al*. [23]. Briefly, a cis-eQTL was determined as an reQTL if this cis-eQTL was present at an FDR of 0.01 in one condition and no signal of cis-eQTL was found in the other condition (FDR > 0.5) among all SNPs tested in the entire 200kb region. An alternative approach to identify reQTL is to treat the fold change gene expression after *M*. *leprae* stimulation as a quantitative trait and map it. Although this approach has the advantage of avoiding arbitrarily selected cut-off points used in our approach, it fails to inform if the genes associated with reQTL show any significant differences in gene expression levels in infected or non-infected cells derived from individual of different genotypes classes. Importantly, it was shown that both approaches provide similar lists of reQTL [23].

### Resampling analysis for testing the enrichment of cis-eQTL and reQTL in GWAS hits

To account for LD between cis-eQTL, we considered only the best eQTL per gene (with the lowest *P* value). We tested whether the leprosy *per se* and T1R GWAS hits (*P* < 0.05) were enriched for cis-eQTL by resampling. We randomly selected 318 variants in each GWAS 10^6^ times and each of the 10^6^ simulated dataset was analyzed and compared with the actual results. The same procedure was performed for the 66 reQTL. In addition to the enrichment analysis of (r)eQTL among GWAS hits, which was measured without taking the frequencies of the tested SNPs into account and only using the best signal for each gene, we also used the GARFIELD software [35]. We performed a more stringent analysis integrating the LD structure between eQTL (or reQTL), their frequencies, and their distance to the nearest TSS.

### Resampling analysis for testing the enrichment of reQTL located in transcription factor binding sites

We extracted the data from HaploReg v4.0 for each of the 1,123 reQTL we identified (all significant association, i.e. not the best signal per gene only), and for all the variants that were tested for being eQTL in our study (i.e. the variants located in the 200kb interval around each gene). We identified which variants were located in a transcription factor binding site (TFBS). We performed a resampling analysis to measure whether reQTL were preferentially located in TFBS. We proceeded as follows: (i) we randomly selected 1,123 variants among the 1,722,978 tested for being cis-eQTL and marked those that were located in a TFBS; (ii) for each transcription factor, we counted the number of variants among the 1,123 randomly selected, that were located in the corresponding binding site. We then repeated steps (i) and (ii) 200,000 times. To complement this approach, we used the SNP2TFBS software, which uses PWM (position weight matrices) and evaluated the effect of SNPs on TFBS (http://ccg.vital-it.ch/snp2tfbs) [33]. This tool also performs an enrichment analysis where the enrichment *P* values are calculated using a binomial distribution of size n = 256 (256 SNPs which matched with at least one TFBS), and probability p which corresponds to the number of SNPs matching a given TF (genome-wide) divided by total number of SNPs matching a TFBS.

### Natural selection analysis

To detect signatures of positive selection acting on the detected cis-eQTL we performed the Derived Intra-allelic Nucleotide Diversity (DIND) on populations from the 1,000 Genomes Project Phase III as previously described [39]. Briefly, we extracted sequence data from the 1,000 Genomes Project Phase III from the 26 populations, including sequence data from the KHV population (Kinh in Ho Chi Minh City, Vietnam) which is the same population as the subjects of our study [37]. Phased sequences were obtained from the MaCH website (Center for Statistical Genetics, University of Michigan) [60]. DIND is a haplotype-based test and is population and SNP-specific [61]. It was designed to highlight variants that are targeted by natural selection. The DIND test has been shown to be robust to demography and virtually insensitive to low depth of coverage generated by next generation sequencing data [39]. The DIND test is based on the iπ_A_/iπ_D_ ratio, where iπ_A_ and iπ_D_ are the levels of nucleotide diversity associated with the haplotypes carrying the ancestral and the derived alleles, respectively. This test was performed on 100kb windows centered on each identified cis-eQTL and normalized [39]. SNPs with derived allele frequencies (DAF) < 20% in the KHV population were excluded from the analysis because of the lack of power to detect signatures of positive selection for the DIND test. *P* values were then obtained by genome-wide ranking. To access the significance of the DIND test, the same procedure described above for the resampling of GWAS hits was performed but on the entire set of variants found in the 1000 genomes project Phase III, with DAF above 20% with increments of 5% frequency per bin.

We also measured the population differentiation levels with the global *F*_ST_ taking into account the 26 populations included in the 1,000 Genomes Project Phase III. Since *F*_ST_ depends exclusively on the frequency of each variant in each population, we stratified the *F*_ST_ values using sliding windows of frequencies of 0.05 with an interval of 0.005 and drew the 95^th^ and 99^th^ percentiles of genome-wide distributions. *F*_ST_ values above the 95^th^ percentile were defined as significant.

## Supporting information

S1 Dataset Genotyping data. DNA from the 51 blood donors were genotyped using Illumina`s Human660W-Quad BeadChip array. After standard filtration methods (no missing data, Hardy-Weinberg *P* value < 10^−4^), 459,703 SNPs were kept for analysis. 3 files are provided in the S1_Datasep.zip archive: Leprosy_eQTL_51_samples.bed, Leprosy_eQTL_51_samples.bim and Leprosy_eQTL_51_samples.fam. These files were generated using PLINK 1.9 (www.cog-genomics.org/plink/1.9/).
(ZIP)

S1 Fig Quantile–quantile plot. QQ-plot of *P* values obtained when testing for an association between gene expression estimates and all SNPs located in a 200-kb window centered on each gene’s transcription starting site (TSS) (y axis) compared with P values obtained by permuting the gene expression measurement (x axis) in non-stimulated cells with 7 PCs removed (pink), and in stimulated cells with 8 PCs removed (blue). An FDR of 0.01 corresponds to observed *P* values < 3.06x10^-6^ in the non-stimulated condition and to *P* values < 1.90x10^-6^ in the stimulated condition.
(TIF)

S2 Fig Principal component analysis to detect population structure. The two first components of a principal component analysis are plotted, including (A) the entire set of the 1000 Genomes Project Phase III sample and the 51 samples of our study, and (B) individuals of East-Asian descent from the 1000 Genomes Project Phase III and the 51 samples of our study. The 51 samples of our study cluster very well with East-Asian samples and in particular with the KHV population. AFR: African; AMR: Ad Mixed American; EAS: East Asian; EUR: European; SAS: South Asian; CDX: Chinese Dai in Xishuangbanna, China; CHB: Han Chinese in Bejing, China; CHS: Southern Han Chinese; JPT: Japanese in Tokyo, Japan; KHV: Kinh in Ho Chi Minh City, Vietnam; LEP: Leprosy patients from our study.
(TIF)

S3 Fig Flowchart summarizing our study. Numbers in parentheses correspond to the number of genes. DEG: Differentially expressed genes passing the Bonferroni correction.
(TIF)

S4 Fig Principal component analysis to identify the effect of covariates on expression data. The two first components of a principal component analysis (PCA) are plotted, to evaluate the effect of (A) the stimulation itself, with unstimulated samples given in blue and stimulated samples in red, (B) age: blue subjects are older than 15 years old, red subjects are younger, (C) gender: women are in blue, men in red, (D) duration of stimulation: samples is blue were stimulated for 32 hours, while samples in red for 26 hours, and (E) dose of *M*. *leprae* antigens samples in red received 20μg/mL of *M*. *leprae* antigens while samples in blue received a lower dose for technical reason. The left panel represents the PCA of the samples obtained from raw expression data, the right panel from adjusted expression data. Expression data were adjusted by keeping only the effects of the stimulation and the residuals from the following multiple regression: Expression ~ Stimulation + Duration of stimulation + Stimulation*Duration of stimulation + Dose + Age + Gender + residuals. The x axis corresponds to the first PC, the y axis to the second PC, labels correspond to the proportion of variance retained by the corresponding PC. Of note, only differences in dose had a noticeable impact, which was corrected successfully by PC adjustment.
(TIF)

S5 Fig Power to detect differentially expressed genes. On the left graph, power to detect differentially expressed genes against fold change is displayed. On the right graph, power to detect a log_2_ (FC) as a function of sample size is plotted.
(TIF)

S1 Table Gene ontology analysis of differentially expressed genes. (XLSX)

S2 Table Details of each identified cis-eQTL. (XLSX)

S3 Table Identified cis-eQTL at an FDR of 0.05. (XLSX)

S4 Table Genes displaying reQTL. (XLSX)

S5 Table Enrichment analysis of reQTL located in binding sites of transcription factors. (XLSX)

S6 Table Enrichment analysis of reQTL disrupting transcription factor binding sites. (XLSX)

S7 Table cis-eQTL targeted by recent positive selection. (XLSX)

## References

[1] Organization WH. Global leprosy update, 2014: need for early case detection. Wkly Epidemiol Rec. 2015;90(36):461–74. . 26343055

[2] Alcais A, Alter A, Antoni G, Orlova M, Nguyen VT, Singh M, et al Stepwise replication identifies a low-producing lymphotoxin-alpha allele as a major risk factor for early-onset leprosy. Nat Genet. 2007;39(4):517–22. doi: 10.1038/ng2000 . 17353895 10.1038/ng2000

[3] Alter A, de Leseleuc L, Van Thuc N, Thai VH, Huong NT, Ba NN, et al Genetic and functional analysis of common MRC1 exon 7 polymorphisms in leprosy susceptibility. Hum Genet. 2010;127(3):337–48. doi: 10.1007/s00439-009-0775-x ; PubMed Central PMCID: PMCPMC2891106. 20035344 10.1007/s00439-009-0775-x PMC2891106

[4] Alter A, Fava VM, Huong NT, Singh M, Orlova M, Van Thuc N, et al Linkage disequilibrium pattern and age-at-diagnosis are critical for replicating genetic associations across ethnic groups in leprosy. Hum Genet. 2013;132(1):107–16. doi: 10.1007/s00439-012-1227-6 . 23052943 10.1007/s00439-012-1227-6

[5] Alter A, Huong NT, Singh M, Orlova M, Van Thuc N, Katoch K, et al Human leukocyte antigen class I region single-nucleotide polymorphisms are associated with leprosy susceptibility in Vietnam and India. J Infect Dis. 2011;203(9):1274–81. doi: 10.1093/infdis/jir024 ; PubMed Central PMCID: PMCPMC3069725. 21459816 10.1093/infdis/jir024 PMC3069725

[6] Cardoso CC, Pereira AC, Brito-de-Souza VN, Duraes SM, Ribeiro-Alves M, Nery JA, et al TNF -308G>A single nucleotide polymorphism is associated with leprosy among Brazilians: a genetic epidemiology assessment, meta-analysis, and functional study. J Infect Dis. 2011;204(8):1256–63. doi: 10.1093/infdis/jir521 . 21917899 10.1093/infdis/jir521

[7] Grant AV, Cobat A, Van Thuc N, Orlova M, Huong NT, Gaschignard J, et al CUBN and NEBL common variants in the chromosome 10p13 linkage region are associated with multibacillary leprosy in Vietnam. Hum Genet. 2014;133(7):883–93. doi: 10.1007/s00439-014-1430-8 . 24563210 10.1007/s00439-014-1430-8

[8] Marques Cde S, Brito-de-Souza VN, Guerreiro LT, Martins JH, Amaral EP, Cardoso CC, et al Toll-like receptor 1 N248S single-nucleotide polymorphism is associated with leprosy risk and regulates immune activation during mycobacterial infection. J Infect Dis. 2013;208(1):120–9. doi: 10.1093/infdis/jit133 . 23547143 10.1093/infdis/jit133

[9] Liu H, Irwanto A, Fu X, Yu G, Yu Y, Sun Y, et al Discovery of six new susceptibility loci and analysis of pleiotropic effects in leprosy. Nat Genet. 2015;47(3):267–71. doi: 10.1038/ng.3212 . 25642632 10.1038/ng.3212

[10] Zhang F, Liu H, Chen S, Low H, Sun L, Cui Y, et al Identification of two new loci at IL23R and RAB32 that influence susceptibility to leprosy. Nat Genet. 2011;43(12):1247–51. doi: 10.1038/ng.973 . 22019778 10.1038/ng.973

[11] Zhang FR, Huang W, Chen SM, Sun LD, Liu H, Li Y, et al Genomewide association study of leprosy. N Engl J Med. 2009;361(27):2609–18. doi: 10.1056/NEJMoa0903753 . 20018961 10.1056/NEJMoa0903753

[12] Abel L, Alcais A, Schurr E. The dissection of complex susceptibility to infectious disease: bacterial, viral and parasitic infections. Curr Opin Immunol. 2014;30:72–8. doi: 10.1016/j.coi.2014.07.002 . 25083600 10.1016/j.coi.2014.07.002

[13] Cobat A, Abel L, Alcais A, Schurr E. A general efficient and flexible approach for genome-wide association analyses of imputed genotypes in family-based designs. Genet Epidemiol. 2014;38(6):560–71. doi: 10.1002/gepi.21842 . 25044438 10.1002/gepi.21842

[14] Grant AV, Alter A, Huong NT, Orlova M, Van Thuc N, Ba NN, et al Crohn's disease susceptibility genes are associated with leprosy in the Vietnamese population. J Infect Dis. 2012;206(11):1763–7. doi: 10.1093/infdis/jis588 . 22984114 10.1093/infdis/jis588

[15] Sales-Marques C, Salomao H, Fava VM, Alvarado-Arnez LE, Amaral EP, Cardoso CC, et al NOD2 and CCDC122-LACC1 genes are associated with leprosy susceptibility in Brazilians. Hum Genet. 2014;133(12):1525–32. doi: 10.1007/s00439-014-1502-9 . 25367361 10.1007/s00439-014-1502-9

[16] Wong SH, Hill AV, Vannberg FO, India-Africa-United Kingdom Leprosy Genetics C. Genomewide association study of leprosy. N Engl J Med. 2010;362(15):1446–7; author reply 7–8. doi: 10.1056/NEJMc1001451 . 20393182 10.1056/NEJMc1001451

[17] Fava VM, Cobat A, Van Thuc N, Latini AC, Stefani MM, Belone AF, et al Association of TNFSF8 regulatory variants with excessive inflammatory responses but not leprosy per se. J Infect Dis. 2015;211(6):968–77. doi: 10.1093/infdis/jiu566 . 25320285 10.1093/infdis/jiu566

[18] Fava VM, Manry J, Cobat A, Orlova M, Van Thuc N, Ba NN, et al A Missense LRRK2 Variant Is a Risk Factor for Excessive Inflammatory Responses in Leprosy. PLoS Negl Trop Dis. 2016;10(2):e0004412 doi: 10.1371/journal.pntd.0004412 ; PubMed Central PMCID: PMCPMC4742274. 26844546 10.1371/journal.pntd.0004412 PMC4742274

[19] Fava VM, Sales-Marques C, Alcais A, Moraes MO, Schurr E. Age-Dependent Association of TNFSF15/TNFSF8 Variants and Leprosy Type 1 Reaction. Front Immunol. 2017;8:155 doi: 10.3389/fimmu.2017.00155 . 28261213 10.3389/fimmu.2017.00155 PMC5306391

[20] Fava VM, Manry J., Cobat A., Orlova M., van Thuc N., Moraes M.O., Schurr E. A Genome Wide Association Study Identifies a lncrna as Risk Factor for Pathological Inflammatory Responses in Leprosy. PloS Genet 2017;13(2):21006637. 10.1371/journal.pgen.1006637 PMC5340414 28222097

[21] Nedelec Y, Sanz J, Baharian G, Szpiech ZA, Pacis A, Dumaine A, et al Genetic Ancestry and Natural Selection Drive Population Differences in Immune Responses to Pathogens. Cell. 2016;167(3):657–69 e21. doi: 10.1016/j.cell.2016.09.025 . 27768889 10.1016/j.cell.2016.09.025

[22] Quach H, Rotival M, Pothlichet J, Loh YE, Dannemann M, Zidane N, et al Genetic Adaptation and Neandertal Admixture Shaped the Immune System of Human Populations. Cell. 2016;167(3):643–56 e17. doi: 10.1016/j.cell.2016.09.024 ; PubMed Central PMCID: PMCPMC5075285. 27768888 10.1016/j.cell.2016.09.024 PMC5075285

[23] Barreiro LB, Tailleux L, Pai AA, Gicquel B, Marioni JC, Gilad Y. Deciphering the genetic architecture of variation in the immune response to Mycobacterium tuberculosis infection. Proc Natl Acad Sci U S A. 2012;109(4):1204–9. doi: 10.1073/pnas.1115761109 ; PubMed Central PMCID: PMCPMC3268270. 22233810 10.1073/pnas.1115761109 PMC3268270

[24] Caliskan M, Baker SW, Gilad Y, Ober C. Host genetic variation influences gene expression response to rhinovirus infection. PLoS Genet. 2015;11(4):e1005111 doi: 10.1371/journal.pgen.1005111 ; PubMed Central PMCID: PMCPMC4395341. 25874939 10.1371/journal.pgen.1005111 PMC4395341

[25] Fairfax BP, Humburg P, Makino S, Naranbhai V, Wong D, Lau E, et al Innate immune activity conditions the effect of regulatory variants upon monocyte gene expression. Science. 2014;343(6175):1246949 doi: 10.1126/science.1246949 ; PubMed Central PMCID: PMCPMC4064786. 24604202 10.1126/science.1246949 PMC4064786

[26] Lee MN, Ye C, Villani AC, Raj T, Li W, Eisenhaure TM, et al Common genetic variants modulate pathogen-sensing responses in human dendritic cells. Science. 2014;343(6175):1246980 doi: 10.1126/science.1246980 ; PubMed Central PMCID: PMCPMC4124741. 24604203 10.1126/science.1246980 PMC4124741

[27] Consortium GT. Human genomics. The Genotype-Tissue Expression (GTEx) pilot analysis: multitissue gene regulation in humans. Science. 2015;348(6235):648–60. doi: 10.1126/science.1262110 ; PubMed Central PMCID: PMCPMC4547484. 25954001 10.1126/science.1262110 PMC4547484

[28] Schramm K, Marzi C, Schurmann C, Carstensen M, Reinmaa E, Biffar R, et al Mapping the genetic architecture of gene regulation in whole blood. PLoS One. 2014;9(4):e93844 doi: 10.1371/journal.pone.0093844 ; PubMed Central PMCID: PMCPMC3989189. 24740359 10.1371/journal.pone.0093844 PMC3989189

[29] Westra HJ, Peters MJ, Esko T, Yaghootkar H, Schurmann C, Kettunen J, et al Systematic identification of trans eQTLs as putative drivers of known disease associations. Nat Genet. 2013;45(10):1238–43. doi: 10.1038/ng.2756 ; PubMed Central PMCID: PMCPMC3991562. 24013639 10.1038/ng.2756 PMC3991562

[30] Fava VM SE. The complexity of the host genetic contribution to the human response to Mycobacterium leprae. The International Textbook of Leprosy 2016.

[31] Storey JD, Tibshirani R. Statistical significance for genomewide studies. Proc Natl Acad Sci U S A. 2003;100(16):9440–5. doi: 10.1073/pnas.1530509100 ; PubMed Central PMCID: PMCPMC170937. 12883005 10.1073/pnas.1530509100 PMC170937

[32] Orlova M, Cobat A, Huong NT, Ba NN, Van Thuc N, Spencer J, et al Gene set signature of reversal reaction type I in leprosy patients. PLoS Genet. 2013;9(7):e1003624 doi: 10.1371/journal.pgen.1003624 ; PubMed Central PMCID: PMCPMC3708838. 23874223 10.1371/journal.pgen.1003624 PMC3708838

[33] Kumar S, Ambrosini G, Bucher P. SNP2TFBS—a database of regulatory SNPs affecting predicted transcription factor binding site affinity. Nucleic Acids Res. 2017;45(D1):D139–D44. doi: 10.1093/nar/gkw1064 ; PubMed Central PMCID: PMCPMC5210548. 27899579 10.1093/nar/gkw1064 PMC5210548

[34] Rhen T, Cidlowski JA. Antiinflammatory action of glucocorticoids—new mechanisms for old drugs. N Engl J Med. 2005;353(16):1711–23. doi: 10.1056/NEJMra050541 . 16236742 10.1056/NEJMra050541

[35] Iotchkova V, Huang J, Morris JA, Jain D, Barbieri C, Walter K, et al Discovery and refinement of genetic loci associated with cardiometabolic risk using dense imputation maps. Nat Genet. 2016;48(11):1303–12. doi: 10.1038/ng.3668 ; PubMed Central PMCID: PMCPMC5279872. 27668658 10.1038/ng.3668 PMC5279872

[36] Kudaravalli S, Veyrieras JB, Stranger BE, Dermitzakis ET, Pritchard JK. Gene expression levels are a target of recent natural selection in the human genome. Mol Biol Evol. 2009;26(3):649–58. doi: 10.1093/molbev/msn289 ; PubMed Central PMCID: PMCPMC2767089. 19091723 10.1093/molbev/msn289 PMC2767089

[37] Sudmant PH, Rausch T, Gardner EJ, Handsaker RE, Abyzov A, Huddleston J, et al An integrated map of structural variation in 2,504 human genomes. Nature. 2015;526(7571):75–81. doi: 10.1038/nature15394 ; PubMed Central PMCID: PMCPMC4617611. 26432246 10.1038/nature15394 PMC4617611

[38] Barreiro LB, Ben-Ali M, Quach H, Laval G, Patin E, Pickrell JK, et al Evolutionary dynamics of human Toll-like receptors and their different contributions to host defense. PLoS Genet. 2009;5(7):e1000562 doi: 10.1371/journal.pgen.1000562 ; PubMed Central PMCID: PMCPMC2702086. 19609346 10.1371/journal.pgen.1000562 PMC2702086

[39] Fagny M, Patin E, Enard D, Barreiro LB, Quintana-Murci L, Laval G. Exploring the occurrence of classic selective sweeps in humans using whole-genome sequencing data sets. Mol Biol Evol. 2014;31(7):1850–68. doi: 10.1093/molbev/msu118 . 24694833 10.1093/molbev/msu118

[40] Manry J, Laval G, Patin E, Fornarino S, Itan Y, Fumagalli M, et al Evolutionary genetic dissection of human interferons. J Exp Med. 2011;208(13):2747–59. doi: 10.1084/jem.20111680 ; PubMed Central PMCID: PMCPMC3244034. 22162829 10.1084/jem.20111680 PMC3244034

[41] Giannakopoulos NV, Arutyunova E, Lai C, Lenschow DJ, Haas AL, Virgin HW. ISG15 Arg151 and the ISG15-conjugating enzyme UbE1L are important for innate immune control of Sindbis virus. J Virol. 2009;83(4):1602–10. doi: 10.1128/JVI.01590-08 ; PubMed Central PMCID: PMCPMC2643764. 19073728 10.1128/JVI.01590-08 PMC2643764

[42] Haas AL, Ahrens P, Bright PM, Ankel H. Interferon induces a 15-kilodalton protein exhibiting marked homology to ubiquitin. J Biol Chem. 1987;262(23):11315–23. . 2440890

[43] Yuan W, Krug RM. Influenza B virus NS1 protein inhibits conjugation of the interferon (IFN)-induced ubiquitin-like ISG15 protein. EMBO J. 2001;20(3):362–71. doi: 10.1093/emboj/20.3.362 ; PubMed Central PMCID: PMCPMC133459. 11157743 10.1093/emboj/20.3.362 PMC133459

[44] Zhao C, Denison C, Huibregtse JM, Gygi S, Krug RM. Human ISG15 conjugation targets both IFN-induced and constitutively expressed proteins functioning in diverse cellular pathways. Proc Natl Acad Sci U S A. 2005;102(29):10200–5. doi: 10.1073/pnas.0504754102 ; PubMed Central PMCID: PMCPMC1177427. 16009940 10.1073/pnas.0504754102 PMC1177427

[45] Bogunovic D, Byun M, Durfee LA, Abhyankar A, Sanal O, Mansouri D, et al Mycobacterial disease and impaired IFN-gamma immunity in humans with inherited ISG15 deficiency. Science. 2012;337(6102):1684–8. doi: 10.1126/science.1224026 ; PubMed Central PMCID: PMCPMC3507439. 22859821 10.1126/science.1224026 PMC3507439

[46] Zhang X, Bogunovic D, Payelle-Brogard B, Francois-Newton V, Speer SD, Yuan C, et al Human intracellular ISG15 prevents interferon-alpha/beta over-amplification and auto-inflammation. Nature. 2015;517(7532):89–93. doi: 10.1038/nature13801 ; PubMed Central PMCID: PMCPMC4303590. 25307056 10.1038/nature13801 PMC4303590

[47] Barreiro LB, Quintana-Murci L. From evolutionary genetics to human immunology: how selection shapes host defence genes. Nat Rev Genet. 2010;11(1):17–30. doi: 10.1038/nrg2698 . 19953080 10.1038/nrg2698

[48] Hamblin MT, Di Rienzo A. Detection of the signature of natural selection in humans: evidence from the Duffy blood group locus. Am J Hum Genet. 2000;66(5):1669–79. doi: 10.1086/302879 ; PubMed Central PMCID: PMCPMC1378024. 10762551 10.1086/302879 PMC1378024

[49] Guerreiro LT, Robottom-Ferreira AB, Ribeiro-Alves M, Toledo-Pinto TG, Rosa Brito T, Rosa PS, et al Gene expression profiling specifies chemokine, mitochondrial and lipid metabolism signatures in leprosy. PLoS One. 2013;8(6):e64748 doi: 10.1371/journal.pone.0064748 ; PubMed Central PMCID: PMCPMC3683049. 23798993 10.1371/journal.pone.0064748 PMC3683049

[50] Blischak JD, Tailleux L, Mitrano A, Barreiro LB, Gilad Y. Mycobacterial infection induces a specific human innate immune response. Sci Rep. 2015;5:16882 doi: 10.1038/srep16882 ; PubMed Central PMCID: PMCPMC4653619. 26586179 10.1038/srep16882 PMC4653619

[51] Du P, Kibbe WA, Lin SM. lumi: a pipeline for processing Illumina microarray. Bioinformatics. 2008;24(13):1547–8. doi: 10.1093/bioinformatics/btn224 . 18467348 10.1093/bioinformatics/btn224

[52] Schurmann C, Heim K, Schillert A, Blankenberg S, Carstensen M, Dorr M, et al Analyzing illumina gene expression microarray data from different tissues: methodological aspects of data analysis in the metaxpress consortium. PLoS One. 2012;7(12):e50938 doi: 10.1371/journal.pone.0050938 ; PubMed Central PMCID: PMCPMC3517598. 23236413 10.1371/journal.pone.0050938 PMC3517598

[53] Jeanmougin M, de Reynies A, Marisa L, Paccard C, Nuel G, Guedj M. Should we abandon the t-test in the analysis of gene expression microarray data: a comparison of variance modeling strategies. PLoS One. 2010;5(9):e12336 doi: 10.1371/journal.pone.0012336 ; PubMed Central PMCID: PMCPMC2933223. 20838429 10.1371/journal.pone.0012336 PMC2933223

[54] Ritchie ME, Phipson B, Wu D, Hu Y, Law CW, Shi W, et al limma powers differential expression analyses for RNA-sequencing and microarray studies. Nucleic Acids Res. 2015;43(7):e47 doi: 10.1093/nar/gkv007 ; PubMed Central PMCID: PMCPMC4402510. 25605792 10.1093/nar/gkv007 PMC4402510

[55] Qiu W, Ting Lee ML, Whitmore GA. sizepower: Sample Size and Power Calculation in Micorarray Studies. 2017.

[56] Eden E, Navon R, Steinfeld I, Lipson D, Yakhini Z. GOrilla: a tool for discovery and visualization of enriched GO terms in ranked gene lists. BMC Bioinformatics. 2009;10:48 doi: 10.1186/1471-2105-10-48 ; PubMed Central PMCID: PMCPMC2644678. 19192299 10.1186/1471-2105-10-48 PMC2644678

[57] Hochberg Y, Benjamini Y. More powerful procedures for multiple significance testing. Stat Med. 1990;9(7):811–8. . 2218183 10.1002/sim.4780090710

[58] Delaneau O, Marchini J, Zagury JF. A linear complexity phasing method for thousands of genomes. Nat Methods. 2011;9(2):179–81. doi: 10.1038/nmeth.1785 . 22138821 10.1038/nmeth.1785

[59] Howie BN, Donnelly P, Marchini J. A flexible and accurate genotype imputation method for the next generation of genome-wide association studies. PLoS Genet. 2009;5(6):e1000529 doi: 10.1371/journal.pgen.1000529 ; PubMed Central PMCID: PMCPMC2689936. 19543373 10.1371/journal.pgen.1000529 PMC2689936

[60] Li Y, Willer CJ, Ding J, Scheet P, Abecasis GR. MaCH: using sequence and genotype data to estimate haplotypes and unobserved genotypes. Genet Epidemiol. 2010;34(8):816–34. doi: 10.1002/gepi.20533 ; PubMed Central PMCID: PMCPMC3175618. 21058334 10.1002/gepi.20533 PMC3175618

[61] Voight BF, Kudaravalli S, Wen X, Pritchard JK. A map of recent positive selection in the human genome. PLoS Biol. 2006;4(3):e72 doi: 10.1371/journal.pbio.0040072 ; PubMed Central PMCID: PMCPMC1382018. 16494531 10.1371/journal.pbio.0040072 PMC1382018

