# Supplementary figures and images for: Deciphering the genetic control of gene expression following Mycobacterium leprae antigen stimulation
Source: PLoS Genet. 2017 Aug 9;13(8):e1006952. doi: 10.1371/journal.pgen.1006952 (PMC5565194; doi:10.1371/journal.pgen.1006952)

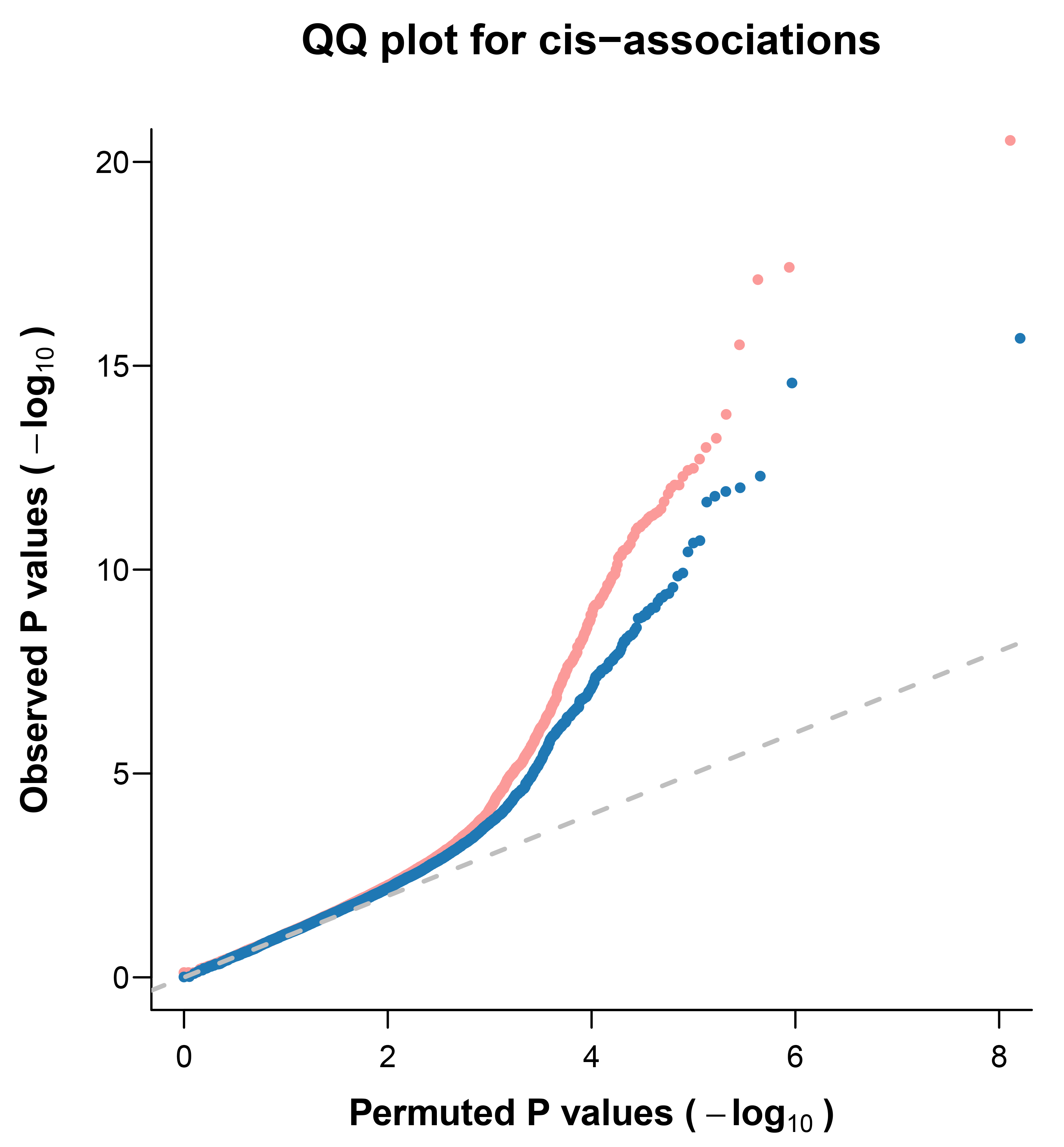

Supplement: S1 Fig — QQ-plot of P values obtained when testing for an association between gene expression estimates and all SNPs located in a 200-kb window centered on each gene’s transcription starting site (TSS) (y axis) compared with P values obtained by permuting the gene expression measurement (x axis) in non-stimulated cells with 7 PCs removed (pink), and in stimulated cells with 8 PCs removed (blue). An FDR of 0.01 corresponds to observed P values < 3.06x10^-6^ in the non-stimulated condition and to P values < 1.90x10^-6^ in the stimulated condition. (TIF) [file pgen.1006952.s002.tif]

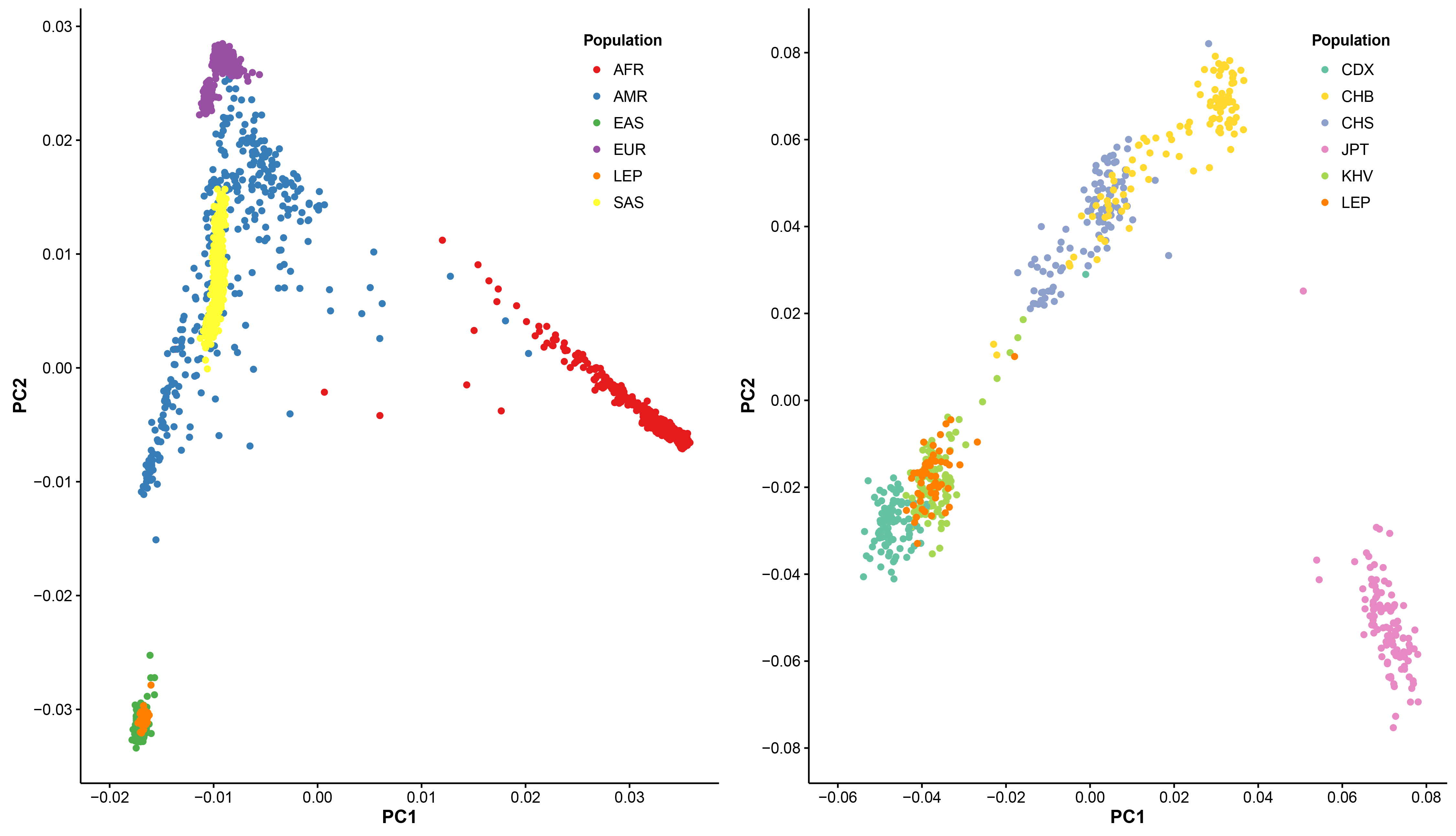

Supplement: S2 Fig — The two first components of a principal component analysis are plotted, including (A) the entire set of the 1000 Genomes Project Phase III sample and the 51 samples of our study, and (B) individuals of East-Asian descent from the 1000 Genomes Project Phase III and the 51 samples of our study. The 51 samples of our study cluster very well with East-Asian samples and in particular with the KHV population. AFR: African; AMR: Ad Mixed American; EAS: East Asian; EUR: European; SAS: South Asian; CDX: Chinese Dai in Xishuangbanna, China; CHB: Han Chinese in Bejing, China; CHS: Southern Han Chinese; JPT: Japanese in Tokyo, Japan; KHV: Kinh in Ho Chi Minh City, Vietnam; LEP: Leprosy patients from our study. (TIF) [file pgen.1006952.s003.tif]

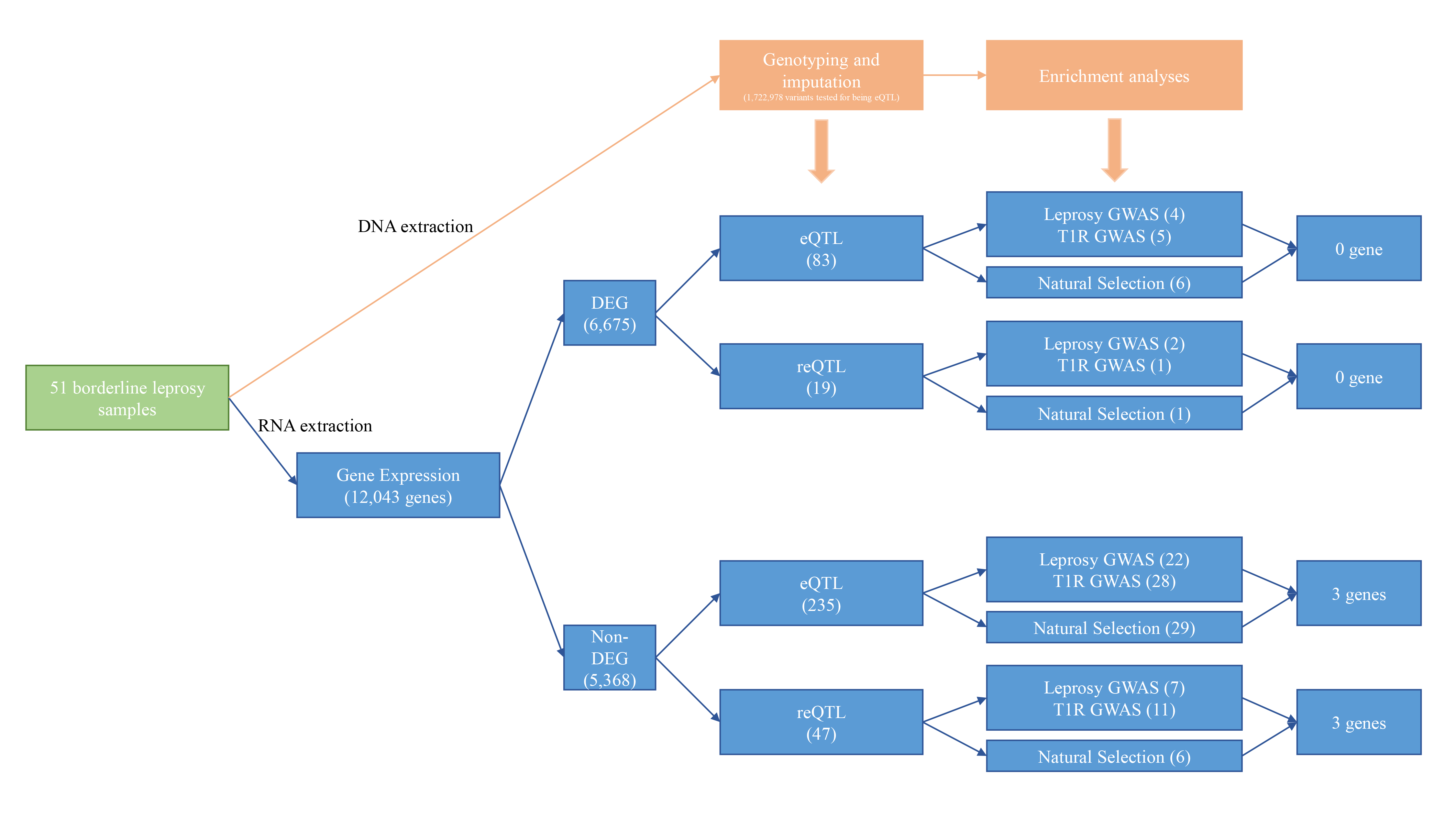

Supplement: S3 Fig — Numbers in parentheses correspond to the number of genes. DEG: Differentially expressed genes passing the Bonferroni correction. (TIF) [file pgen.1006952.s004.tif]

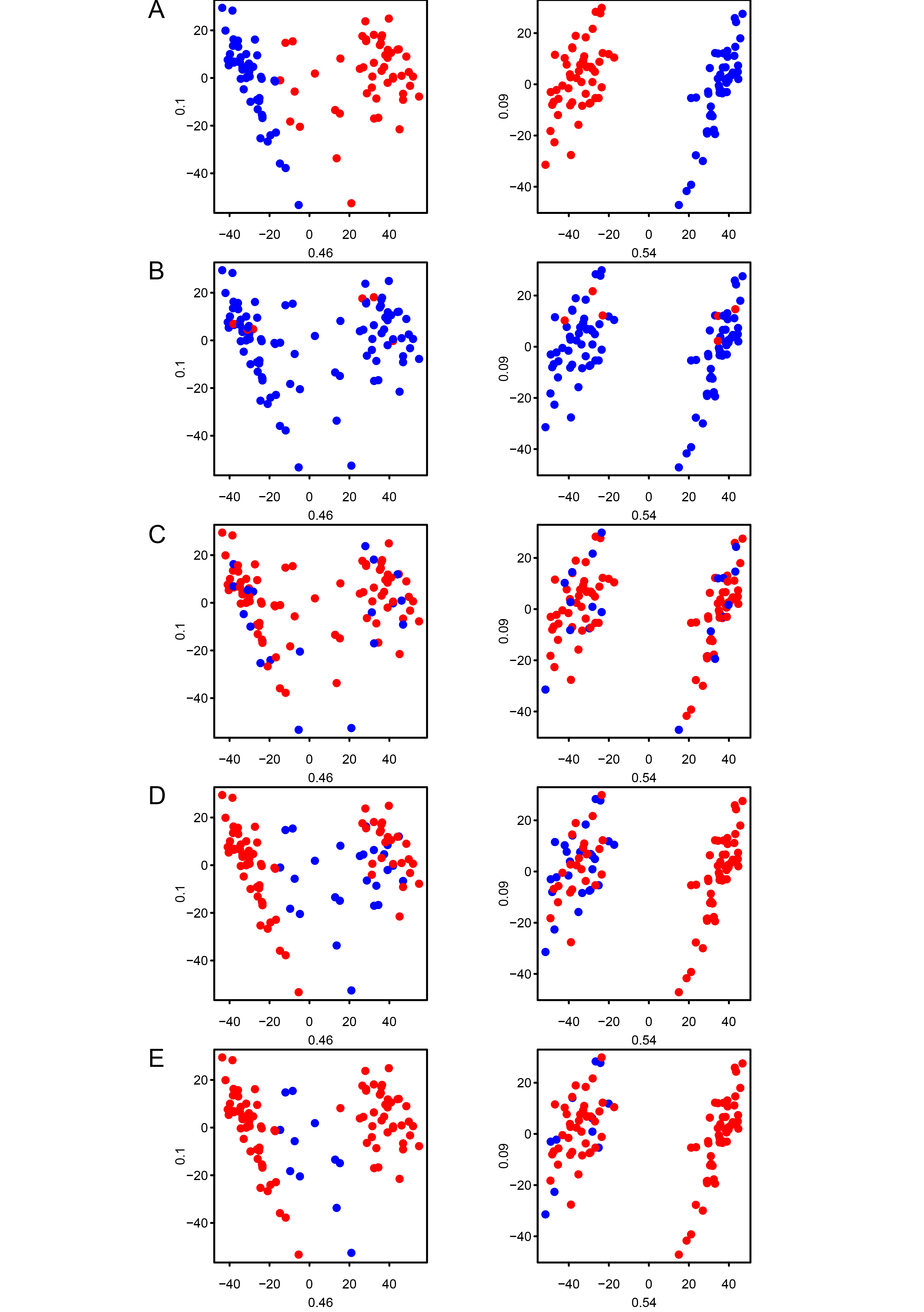

Supplement: S4 Fig — The two first components of a principal component analysis (PCA) are plotted, to evaluate the effect of (A) the stimulation itself, with unstimulated samples given in blue and stimulated samples in red, (B) age: blue subjects are older than 15 years old, red subjects are younger, (C) gender: women are in blue, men in red, (D) duration of stimulation: samples is blue were stimulated for 32 hours, while samples in red for 26 hours, and (E) dose of M. leprae antigens samples in red received 20μg/mL of M. leprae antigens while samples in blue received a lower dose for technical reason. The left panel represents the PCA of the samples obtained from raw expression data, the right panel from adjusted expression data. Expression data were adjusted by keeping only the effects of the stimulation and the residuals from the following multiple regression: Expression ~ Stimulation + Duration of stimulation + Stimulation*Duration of stimulation + Dose + Age + Gender + residuals. The x axis corresponds to the first PC, the y axis to the second PC, labels correspond to the proportion of variance retained by the corresponding PC. Of note, only differences in dose had a noticeable impact, which was corrected successfully by PC adjustment. (TIF) [file pgen.1006952.s005.tif]

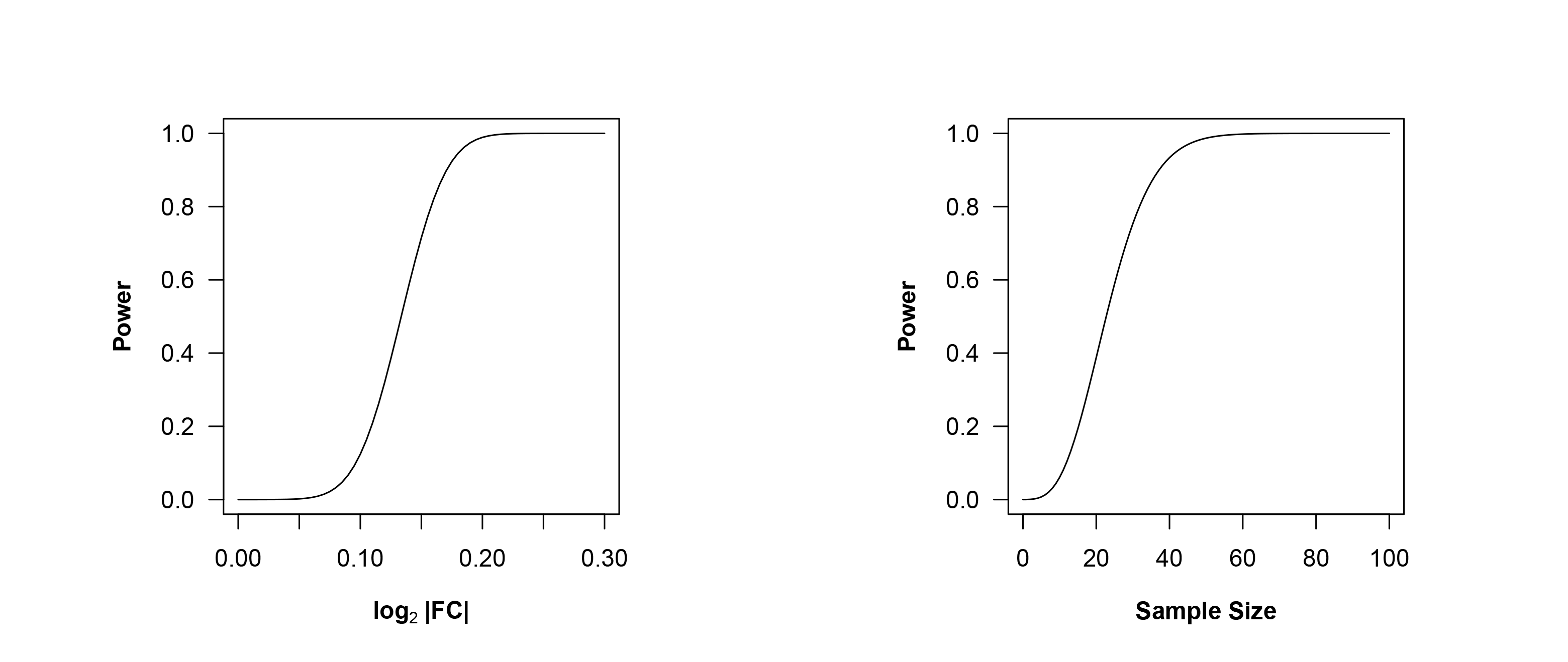

Supplement: S5 Fig — On the left graph, power to detect differentially expressed genes against fold change is displayed. On the right graph, power to detect a log2 (FC) as a function of sample size is plotted. (TIF) [file pgen.1006952.s006.tif]
